# Supplementary material for: No universal mathematical model for thermal performance curves across traits and taxonomic groups
Source: Nat Commun. 2024 Oct 14;15:8855. doi: 10.1038/s41467-024-53046-2 (PMC11473535; doi:10.1038/s41467-024-53046-2)
Supplement: Supplementary file 1 — Supplementary Information [file 41467_2024_53046_MOESM1_ESM.pdf]

Supplementary Information for

No universal mathematical model for thermal performance curves across traits and taxonomic groups

Dimitrios - Georgios Kontopoulos[1,2,3,✉], Arnaud Sentis[4], Martin Daufresne[4], Natalia Glazman[1], Anthony I. Dell[5,6], & Samraat Pawar[1]

[1]Department of Life Sciences, Imperial College London, Silwood Park, Buckhurst Road, Ascot, Berkshire, SL5 7PY, UK

[2]LOEWE Centre for Translational Biodiversity Genomics, Senckenberganlage 25, 60325, Frankfurt, Germany

[3]Senckenberg Research Institute, Senckenberganlage 25, 60325, Frankfurt, Germany

[4]INRAE, Aix Marseille University, UMR RECOVER, 3275 Route de Cézanne - CS 40061, 13182 Aix-en-Provence Cedex 5, France

[5]National Great Rivers Research and Education Center, One Confluence Way, 62024, East Alton, Illinois, USA

[6]Department of Biology, Washington University in St. Louis, 1 Brookings Drive, 63130, St. Louis, Missouri, USA

✉email: dgkontopoulos@gmail.com

Contents

Supplementary Note 1: Models and thermal performance datasets examined in previous studies 2

Supplementary Note 2: Model fits included in the present study 4

Supplementary Note 3: Comparison of the ten best-performing models across traits 6

Supplementary Note 4: Comparison of model performance across datasets of individual enzymes 7

Supplementary Note 5: TPC descriptor variables 8

Supplementary Note 6: The topology of the final conditional inference tree 11

Supplementary Note 7: Thermal performance curve models included in this study 12

Supplementary Note 1: Models and thermal performance datasets examined in previous studies

Supplementary Table 1: Description of previous studies that compared the performance of alternative TPC models across one or more thermal performance datasets.

Study	Taxonomic breadth	Trait(s)	Model count	Dataset count
Angilletta Jr, M. J. Estimating and comparing thermal performance curves. *J. Therm. Biol.* **31**, 541–545 (2006).	*Sceloporus undulatus* (eastern fence lizard)	maximal sprint speed	5	1
Shi, P. & Ge, F. A comparison of different thermal performance functions describing temperature-dependent development rates. *J. Therm. Biol.* **35**, 225–231 (2010).	*Plutella xylostella* (diamondback moth) and *Bemisia tabaci* (silverleaf whitefly)	development rate	12	2
Krenek, S., Berendonk, T. U. & Petzoldt, T. Thermal performance curves of Paramecium caudatum: a model selection approach. *Eur. J. Protistol.* **47**, 124–137 (2011).	*Paramecium caudatum* (a unicellular protist)	population growth rate	12	4
Shi, P.-J., Reddy, G. V., Chen, L. & Ge, F. Comparison of thermal performance equations in describing temperature-dependent developmental rates of insects: (I) empirical models. *Ann. Entomol. Soc. Am.* **109**, 211–215 (2016).	9 insect species and *Kampimodromus aberrans* (a mite)	development rate	6	10

Supplementary Table 1 – *Continued from previous page*

Shi, P.-J., Reddy, G. V., Chen, L. & Ge, F. Comparison of thermal performance equations in describing temperature-dependent developmental rates of insects: (II) two thermodynamic models. *Ann. Entomol. Soc. Am.* **110**, 113–120 (2017).	9 insect species and *Kampimodromus aberrans* (a mite)	development rate	2	10
Low-Décarie, E. et al. Predictions of response to temperature are contingent on model choice and data quality. *Ecol. Evol.* **7**, 10467–10481 (2017).	mainly phytoplankton and fungi, but also bacteria, ants, and others	mainly population growth rate, but also photosynthesis, filtration rate, and others	12	381
Quinn, B. K. Performance of the SSI development function compared with 33 other functions applied to 79 arthropod species' datasets *J. Therm. Biol.* **102**, 103112 (2021).	arthropods	development rate	34	79

Supplementary Note 2: Model fits included in the present study

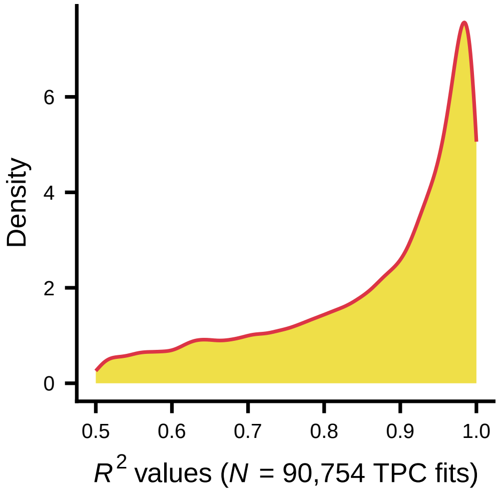

Supplementary Fig. 1: R^2 values for all TPC model fits that passed our filtering criteria (see Methods in the main text). The distribution is heavily left-skewed, indicating that most model fits that were included in this study were able to accurately represent the underlying measurements of thermal performance. Source data are provided as a Source Data file.

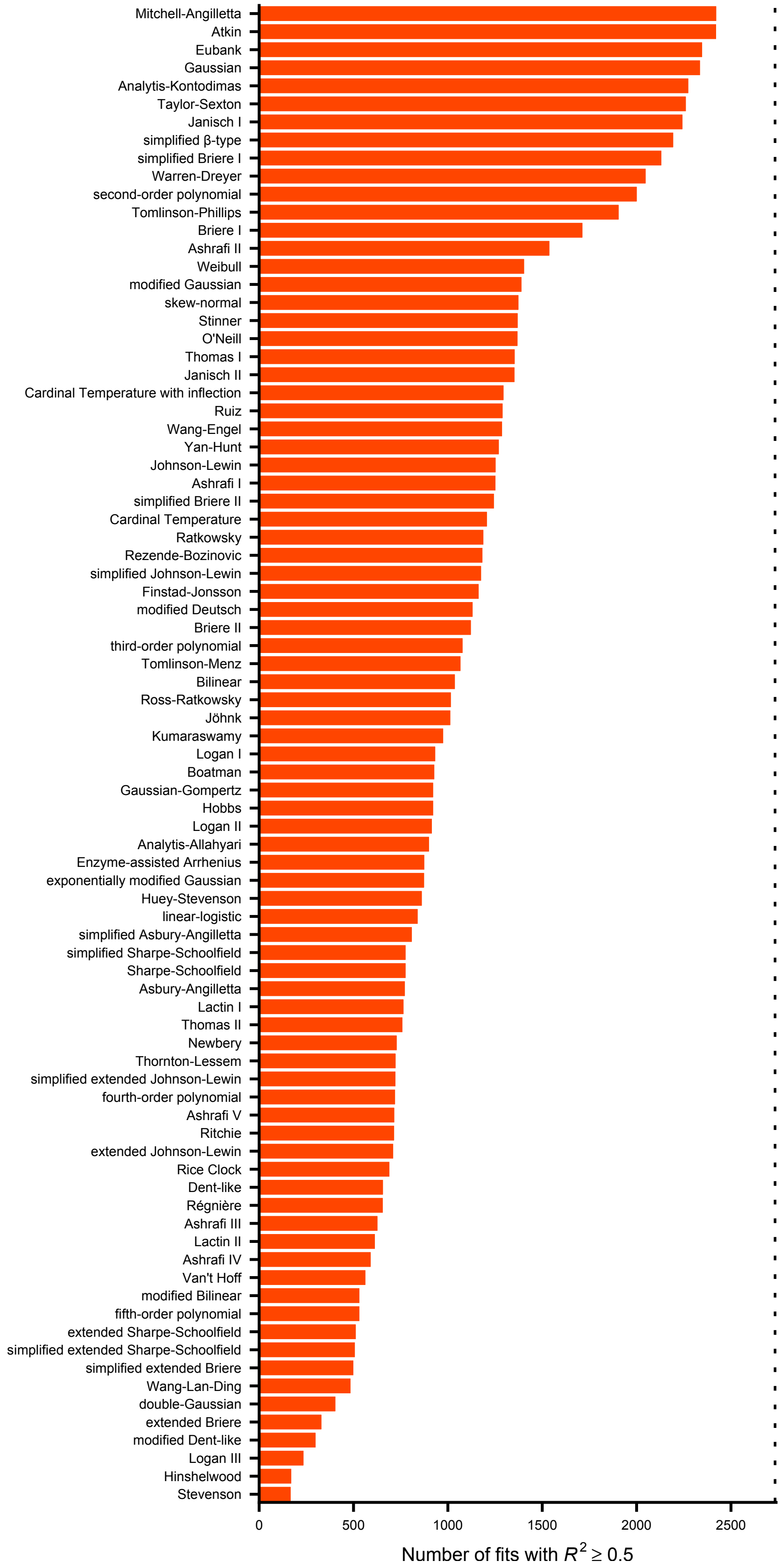

Supplementary Fig. 2: The number of fits per model in our study. The dotted line stands for the total number of thermal performance datasets that could be fitted well by at least one model (see Methods in the main text). Source data are provided as a Source Data file.

Supplementary Note 3: Comparison of the ten best-performing models across traits

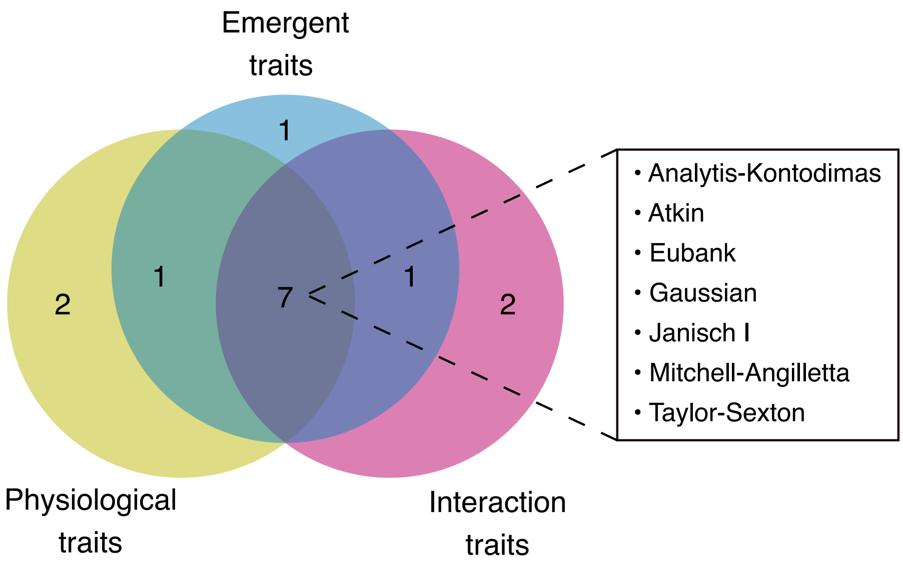

Supplementary Fig. 3: Venn diagram of the ten best-performing models (based on their median AICc weights) across thermal performance datasets of physiological, emergent, and interaction traits. The box shows the models that are among the ten best-performing in all three groups.

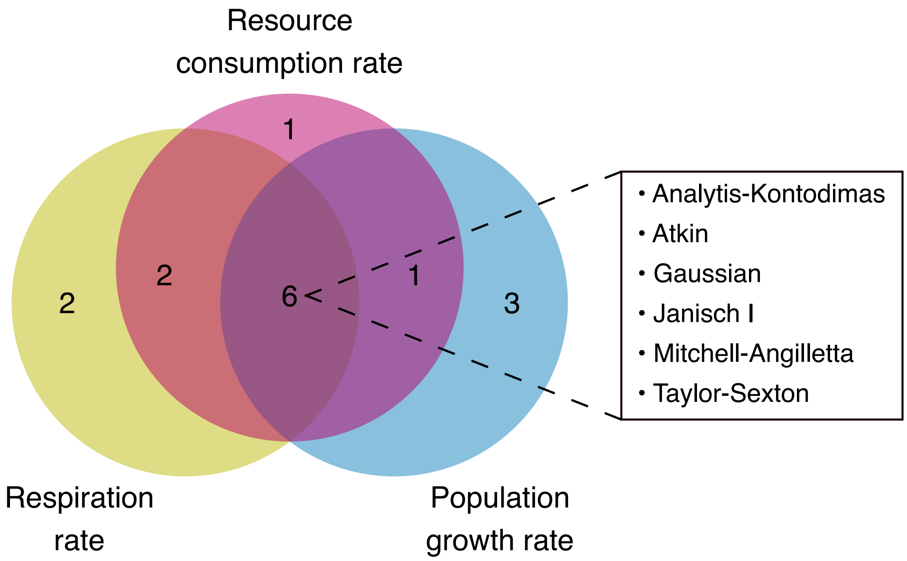

Supplementary Fig. 4: Venn diagram of the ten best-performing models (based on their median AICc weights) across thermal performance datasets of respiration rate, population growth rate, and resource consumption rate. The box shows the models that are among the ten best-performing in all three groups.

Supplementary Note 4: Comparison of model performance across datasets of individual enzymes

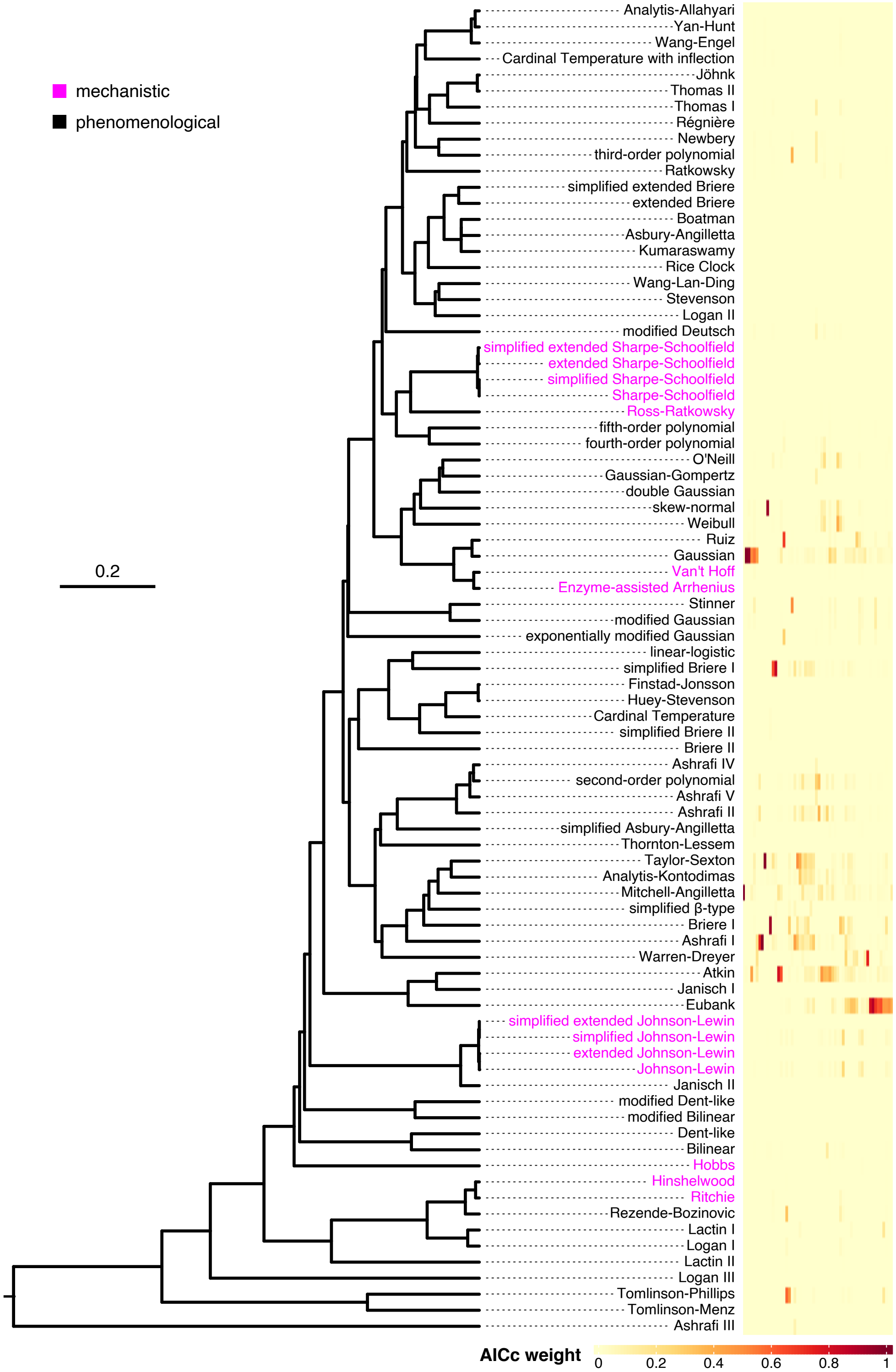

Supplementary Fig. 5: Comparison of the performance of our 83 TPC models across 56 thermal performance datasets of individual enzymes. The dendrogram of models was copied from Fig. 3 in the main text. Source data are provided as a Source Data file.

Supplementary Note 5: TPC descriptor variables

Supplementary Table 2: Names and descriptions of the 29 variables used as possible predictors in the multi-output conditional inference regression trees fitted in the present study. The colour of each row stands for variable type. Variables related to sampling resolution are shown in blue, those describing the shape of the TPC in orange, taxonomic variables in grey-green, and trait information variables in pink.

Variable name	Variable description
`n_data_points`	The number of data points across the entire thermal performance dataset.
`n_data_points_rise`	As `n_data_points`, but below the thermal optimum. The latter was estimated through a regression of trait measurements against the second order polynomial of temperature.<br><br>We considered a thermal performance dataset to have an estimable thermal optimum if the leading coefficient of the polynomial was negative and the p-value of the regression was < 0.05.<br><br>If either of these conditions was not met, we estimated the correlation between trait measurements and temperatures. A statistically significant positive/negative correlation would indicate that trait measurements are rising/falling throughout the sampled temperature range.<br><br>If the correlation was not statistically significant, we could not confidently classify the trait measurements as rising, falling, or unimodal and, thus, we assigned NA to this variable.
`n_data_points_fall`	As `n_data_points`, but above the thermal optimum.
`n_distinct_temperatures`	The number of distinct temperatures across the entire thermal performance dataset.
`n_distinct_temperatures_rise`	As `n_distinct_temperatures`, but below the thermal optimum.

`n_distinct_temperatures_fall`	As `n_distinct_temperatures`, but above the thermal optimum.
`correlation_rise`	The correlation between trait measurements and temperatures below the thermal optimum. It is a metric of measurement noise.
`correlation_fall`	As `correlation_rise`, but above the thermal optimum.
`temperature_range_width`	The difference between the maximum and minimum experimental temperatures across the entire thermal performance dataset.
`temperature_range_width_rise`	As `temperature_range_width`, but below the thermal optimum.
`temperature_range_width_fall`	As `temperature_range_width`, but above the thermal optimum.
`median_distance_between_temperatures`	The median distance between consecutive temperatures across the entire thermal performance dataset.
`median_distance_between_temperatures_rise`	As `median_distance_between_temperatures`, but below the thermal optimum.
`median_distance_between_temperatures_fall`	As `median_distance_between_temperatures`, but above the thermal optimum.
`median_distance_between_measurements`	The median distance between trait measurements at consecutive temperatures across the entire thermal performance dataset, divided by the range of trait measurements.
`median_distance_between_measurements_rise`	As `median_distance_between_measurements`, but below the thermal optimum.
`median_distance_between_measurements_fall`	As `median_distance_between_measurements`, but above the thermal optimum.
`skew_scalar`	The skew scalar (λ) parameter estimate of the skew-normal model (see Supplementary Section S4). Negative values of λ correspond to negatively-skewed TPCs, and vice versa.
`minimum_temperature`	The minimum experimental temperature.

Supplementary Table 2 – *Continued from previous page*

maximum_temperature	The maximum experimental temperature.
thermal_optimum	See n_data_points_rise.
temperature_exponent_rise	Assuming that trait measurements below the thermal optimum increase as a power law of temperature, this variable stands for the exponent of temperature. It is a metric of how steeply the TPC rises up to its peak.
temperature_exponent_fall	As temperature_exponent_rise, but above the thermal optimum.
minimum_to_maximum_measurement_rise	The ratio of the minimum trait measurement below the thermal optimum over the maximum trait measurement.
minimum_to_maximum_measurement_fall	As minimum_to_maximum_measurement_rise, but above the thermal optimum.
kingdom	The kingdom to which the organism belongs.
phylum	The phylum to which the organism belongs.
trait_name	The name of the trait that was measured.
trait_group	The type of the trait that was measured (physiological, emergent, or interaction).

Supplementary Note 6: The topology of the final conditional inference tree

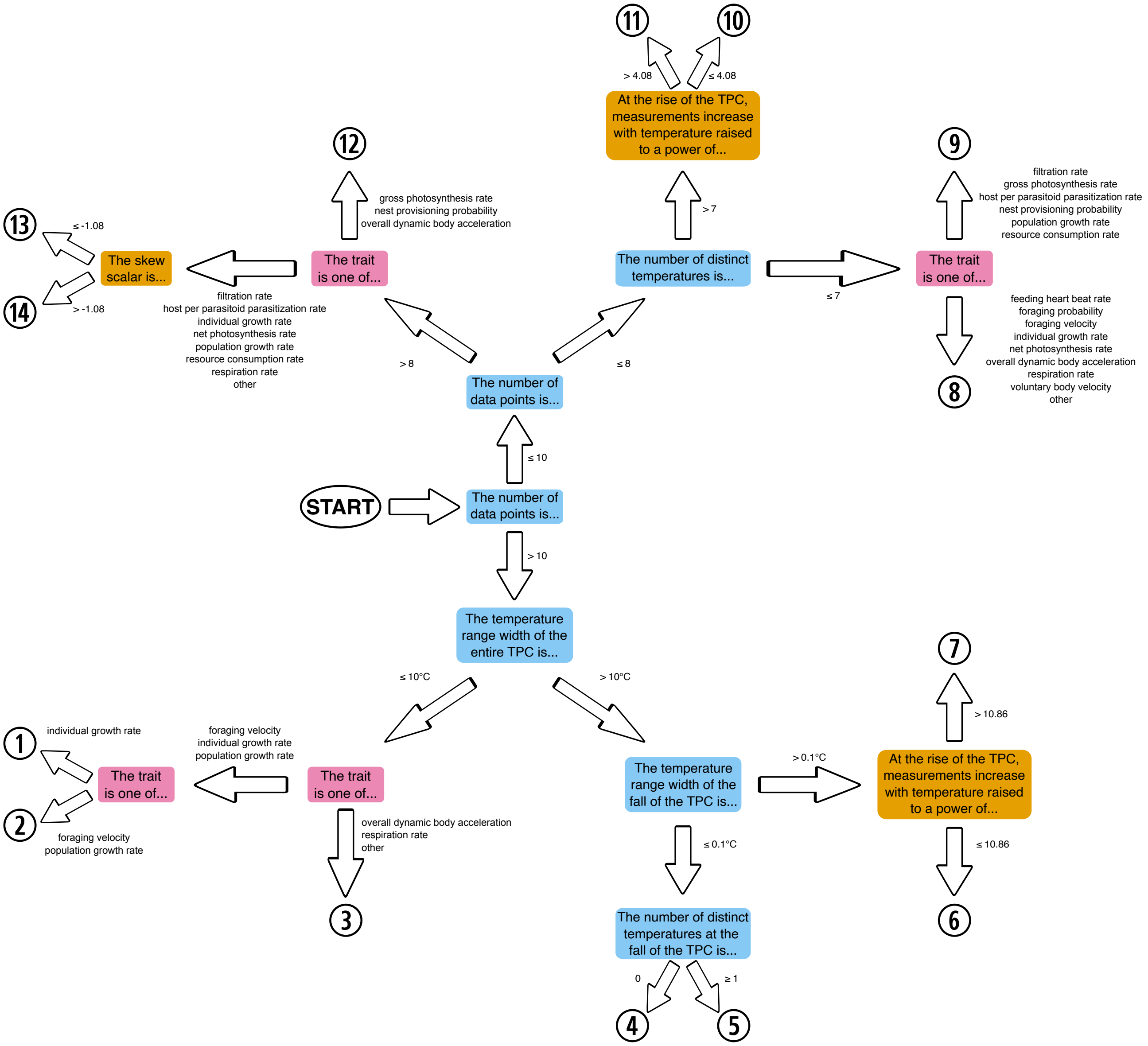

Supplementary Fig. 6: The best-fitting conditional inference tree, based on the R^2 value obtained across the training data subset. Internal nodes are coloured according to variable type. Blue/orange/pink nodes represent sampling resolution, TPC shape, and trait identity variables, respectively. Circled numbers correspond to the leaf nodes of the tree (i.e., thermal performance datasets with distinct patterns of AICc weights).

Supplementary Note 7: Thermal performance curve models included in this study

The varying parameters of each model are shown in orange, whereas temperature (T), functions, and constants are shown in black.

1. Analytis-Allahyari (5 parameters)[1]

$$B(T) = a \cdot \left(\frac{T - T_{\mathrm{min}}}{T_{\mathrm{max}} - T_{\mathrm{min}}}\right)^{b} \cdot \left[1 - \left(\frac{T - T_{\mathrm{min}}}{T_{\mathrm{max}} - T_{\mathrm{min}}}\right)^{d}\right]$$

2. Analytis-Kontodimas (3 parameters)[2]

$$B(T) = a \cdot (T - T_{\mathrm{min}})^2 \cdot (T_{\mathrm{max}} - T)$$

3. Asbury-Angilletta (6 parameters)[3]

$$B(T) = \psi \cdot \frac{\left(\frac{T-a}{d}\right)^{(c/b)-1} \cdot \left(1 - \frac{T-a}{d}\right)^{[(1-c)/b]-1} \cdot \Gamma\left(\frac{1}{b}\right)}{\Gamma\left(\frac{c}{b}\right) \cdot \Gamma\left(\frac{1-c}{b}\right)} \cdot \exp\left(\frac{-E}{k_{\mathrm{B}} \cdot T}\right)$$

4. Simplified Asbury-Angilletta (4 parameters)[3]

$$B(T) = \frac{\left(\frac{T-a}{d}\right)^{(c/b)-1} \cdot \left(1 - \frac{T-a}{d}\right)^{[(1-c)/b]-1} \cdot \Gamma\left(\frac{1}{b}\right)}{\Gamma\left(\frac{c}{b}\right) \cdot \Gamma\left(\frac{1-c}{b}\right)}$$

5. Ashrafi I (3 parameters)[4]

$$B(T) = a + b \cdot T^2 \cdot \ln T + c \cdot T^3$$

6. Ashrafi II (3 parameters)[4]

$$B(T) = a + b \cdot T^{3/2} + c \cdot T^2$$

7. Ashrafi III (3 parameters)[4]

$$B(T) = \frac{1}{a + b \cdot \exp(T) + c \cdot \exp(-T)}$$

8. Ashrafi IV (4 parameters)[4]

$$B(T) = a + b \cdot T + c \cdot (\ln T)^2 + d \cdot \sqrt{T}$$

9. Ashrafi V (4 parameters)[4]

$$B(T) = a + b \cdot (\ln T)^2 + c \cdot \ln T + \frac{d \cdot \ln T}{T}$$

10. Atkin (3 parameters)[5]

$$B(T) = B_0 \cdot (a - b \cdot T)^{T/10}$$

11. Bilinear (4 parameters)[6]

$$B(T) = \begin{cases} B_{\mathrm{pk}} \cdot \dfrac{T - T_{\mathrm{min}}}{T_{\mathrm{pk}} - T_{\mathrm{min}}} & \text{for } T_{\mathrm{min}} < T \leq T_{\mathrm{pk}} \\[2ex] B_{\mathrm{pk}} \cdot \dfrac{T_{\mathrm{max}} - T}{T_{\mathrm{max}} - T_{\mathrm{pk}}} & \text{for } T_{\mathrm{pk}} < T < T_{\mathrm{max}} \end{cases}$$

12. Modified bilinear (6 parameters)[7]

$$B(T) = \begin{cases} B_{\text{pk}} \cdot \left(\dfrac{T - T_{\text{min}}}{T_{\text{pk}} - T_{\text{min}}} \right)^{a} & \text{for } T_{\text{min}} < T \leq T_{\text{pk}} \\[2ex] B_{\text{pk}} \cdot \left(\dfrac{T_{\text{max}} - T}{T_{\text{max}} - T_{\text{pk}}} \right)^{b} & \text{for } T_{\text{pk}} < T < T_{\text{max}} \end{cases}$$

13. Boatman (5 parameters)[8]

$$B(T) = B_{\text{pk}} \cdot \left[\sin\left(\pi \cdot \left(\frac{T - T_{\text{min}}}{T_{\text{max}} - T_{\text{min}}} \right)^{\theta} \right) \right]^{\Phi}$$

14. Briere I (3 parameters)[9]

$$B(T) = a \cdot T \cdot (T - T_{\text{min}}) \cdot \sqrt{T_{\text{max}} - T}$$

15. Simplified Briere I (3 parameters)[9]

$$B(T) = a \cdot (T - T_{\text{min}}) \cdot \sqrt{T_{\text{max}} - T}$$

16. Briere II (4 parameters)[9]

$$B(T) = a \cdot T \cdot (T - T_{\text{min}}) \cdot (T_{\text{max}} - T)^{1/b}$$

17. Simplified Briere II (4 parameters)[9]

$$B(T) = a \cdot (T - T_{\text{min}}) \cdot (T_{\text{max}} - T)^{1/b}$$

18. Extended Briere (5 parameters)[9,10]

$$B(T) = a \cdot T \cdot (T - T_{\text{min}})^b \cdot (T_{\text{max}} - T)^c$$

19. Simplified extended Briere (5 parameters)[9,10]

$$B(T) = a \cdot (T - T_{\text{min}})^b \cdot (T_{\text{max}} - T)^c$$

20. Cardinal Temperature (4 parameters)[11]

$$B(T) = B_{\text{pk}} \cdot \left(1 - \frac{(T - T_{\text{pk}})^2}{(T - T_{\text{pk}})^2 + T \cdot (T_{\text{max}} + T_{\text{min}} - T) - T_{\text{max}} \cdot T_{\text{min}}}\right)$$

21. Cardinal Temperature with Inflection (4 parameters)[12]

$$B(T) = B_{\text{pk}} \cdot \frac{(T - T_{\text{max}}) \cdot (T - T_{\text{min}})^2}{(T_{\text{pk}} - T_{\text{min}}) \cdot [(T_{\text{pk}} - T_{\text{min}}) \cdot (T - T_{\text{pk}}) - (T_{\text{pk}} - T_{\text{max}}) \cdot (T_{\text{pk}} + T_{\text{min}} - 2 \cdot T)]}$$

22. Dent-like (5 parameters)[13]

$$B(T) = \begin{cases} B_{\text{pk}} \cdot \dfrac{T - T_{\text{min}}}{T_{\text{pk (l)}} - T_{\text{min}}} & \text{for } T_{\text{min}} < T < T_{\text{pk (l)}} \\ B_{\text{pk}} & \text{for } T_{\text{pk (l)}} \leq T \leq T_{\text{pk (u)}} \\ B_{\text{pk}} \cdot \dfrac{T_{\text{max}} - T}{T_{\text{max}} - T_{\text{pk (u)}}} & \text{for } T_{\text{pk (u)}} < T < T_{\text{max}} \end{cases}$$

23. Modified dent-like (7 parameters)[7]

$$B(T) = \begin{cases} B_{\mathrm{pk}} \cdot \left(\dfrac{T - T_{\mathrm{min}}}{T_{\mathrm{pk\ (l)}} - T_{\mathrm{min}}} \right)^{a} & \text{for } T_{\mathrm{min}} < T < T_{\mathrm{pk\ (l)}} \\ B_{\mathrm{pk}} & \text{for } T_{\mathrm{pk\ (l)}} \leq T \leq T_{\mathrm{pk\ (u)}} \\ B_{\mathrm{pk}} \cdot \left(\dfrac{T_{\mathrm{max}} - T}{T_{\mathrm{max}} - T_{\mathrm{pk\ (u)}}} \right)^{b} & \text{for } T_{\mathrm{pk\ (u)}} < T < T_{\mathrm{max}} \end{cases}$$

24. Modified Deutsch (4 parameters)[14,15]

$$B(T) = \begin{cases} B_{\mathrm{pk}} \cdot \exp\left[- \left(\dfrac{T - T_{\mathrm{pk}}}{2 \cdot \sigma_{\mathrm{p}}} \right)^2 \right] & \text{for } T \leq T_{\mathrm{pk}} \\ B_{\mathrm{pk}} - B_{\mathrm{pk}} \cdot \left(\dfrac{T - T_{\mathrm{pk}}}{T_{\mathrm{pk}} - T_{\mathrm{max}}} \right)^2 & \text{for } T > T_{\mathrm{pk}} \end{cases}$$

25. Enzyme-assisted Arrhenius (5 parameters)[16]

$$B(T) = a \cdot \exp\left[- \frac{E_{\mathrm{b}} - \left(E_{\Delta\mathrm{H}} \cdot \left(1 - \dfrac{T}{T_{\mathrm{m}}} \right) + E_{\Delta\mathrm{C_p}} \cdot \left(T - T_{\mathrm{m}} - T \cdot \ln \dfrac{T}{T_{\mathrm{m}}} \right) \right)}{k_{\mathrm{B}} \cdot T} \right]$$

26. Eubank (3 parameters)[17]

$$B(T) = \frac{a}{(T - T_{\mathrm{pk}})^2 + b}$$

27. Finstad-Jonsson (4 parameters)[18]

$$B(T) = a \cdot (T - T_{\mathrm{min}}) \cdot [1 - \exp(b \cdot (T - T_{\mathrm{max}}))]$$

28. Gaussian (3 parameters)[19]

$$B(T) = B_{\mathrm{pk}} \cdot \exp\left[-0.5 \cdot \left(\frac{|T - T_{\mathrm{pk}}|}{a}\right)^2\right]$$

29. Double Gaussian (4 parameters)[20]

$$B(T) = \begin{cases} B_{\mathrm{pk}}{}^{\mathrm{e} - \dfrac{-(T - T_{\mathrm{pk}})^2}{2 \cdot a^2}} & \text{for } T < T_{\mathrm{pk}} \\ B_{\mathrm{pk}}{}^{\mathrm{e} - \dfrac{-(T - T_{\mathrm{pk}})^2}{2 \cdot (a \cdot b)^2}} & \text{for } T \geq T_{\mathrm{pk}} \end{cases}$$

30. Modified Gaussian (4 parameters)[19]

$$B(T) = B_{\mathrm{pk}} \cdot \exp\left[-0.5 \cdot \left(\frac{|T - T_{\mathrm{pk}}|}{a}\right)^b\right]$$

31. Exponentially modified Gaussian (5 parameters)[19,21]

$$B(T) = \frac{a \cdot c \cdot \sqrt{2 \cdot \pi}}{2 \cdot d} \cdot \exp\left(\frac{b - T}{d} + \frac{c^2}{2 \cdot d^2}\right) \cdot \left[\frac{d}{|d|} - \mathrm{erf}\left(\frac{b - T}{\sqrt{2} \cdot c} + \frac{c}{\sqrt{2} \cdot d}\right)\right] + f$$

32. Gaussian-Gompertz (5 parameters)[22]

$$B(T) = a \cdot \exp\left[-\exp\left(b \cdot (T - T_{\mathrm{pk}}) - \theta\right) - c \cdot (T - T_{\mathrm{pk}})^2\right]$$

33. Hinshelwood (4 parameters)[23]

$$B(T) = a \cdot \exp\left(\frac{-E_1}{R \cdot T}\right) - b \cdot \exp\left(\frac{-E_2}{R \cdot T}\right)$$

34. Hobbs (4 parameters)[24]

$$B(T) = a \cdot \frac{k_{\mathrm{B}} \cdot T}{h} \cdot \exp\left[-\frac{\Delta H^{\ddagger}_{T_{\mathrm{ref}}} + \Delta C^{\ddagger}_{\mathrm{p}} \cdot (T - T_{\mathrm{ref}})}{R \cdot T} + \frac{\Delta S^{\ddagger}_{T_{\mathrm{ref}}} + \Delta C^{\ddagger}_{\mathrm{p}} \cdot \ln(T/T_{\mathrm{ref}})}{R}\right]$$

35. Huey-Stevenson (5 parameters)[25]

$$B(T) = a \cdot [1 - \exp(-b \cdot (T - T_{\mathrm{min}}))] \cdot [1 - \exp(c \cdot (T - T_{\mathrm{max}}))]$$

36. Janisch I (3 parameters)[26]

$$B(T) = \frac{1}{\frac{m}{2} \cdot \left[a^{T - T_{\mathrm{pk}}} + a^{-(T - T_{\mathrm{pk}})}\right]}$$

37. Janisch II (4 parameters)[26]

$$B(T) = \frac{1}{\frac{m}{2} \cdot \left[a^{T - T_{\mathrm{pk}}} + b^{-(T - T_{\mathrm{pk}})}\right]}$$

38. Jöhnk (5 parameters)[27]

$$B(T) = B_{\mathrm{pk}} \cdot \left[1 + a \cdot \left(\left(b^{T-T_{\mathrm{pk}}} - 1\right) - \frac{\ln b}{\ln c} \cdot \left(c^{T-T_{\mathrm{pk}}} - 1\right)\right)\right]$$

39. Johnson-Lewin (4 parameters)[28,29]

$$B(T) = B_0 \cdot T \cdot \frac{\exp\left(\frac{-E}{k_{\mathrm{B}}} \cdot \frac{1}{T}\right)}{1 + \frac{E}{E_{\mathrm{D}} - E} \cdot \exp\left[\frac{E_{\mathrm{D}}}{k_{\mathrm{B}}} \cdot \left(\frac{1}{T_{\mathrm{pk}}} - \frac{1}{T}\right)\right]}$$

40. Extended Johnson-Lewin (5 parameters)[28–30]

$$B(T) = B_0 \cdot T \cdot \frac{\exp\left(\frac{-E}{k_{\mathrm{B}}} \cdot \frac{1}{T}\right)}{a + \frac{E}{E_{\mathrm{D}} - E} \cdot \exp\left[\frac{E_{\mathrm{D}}}{k_{\mathrm{B}}} \cdot \left(\frac{1}{T_{\mathrm{pk}}} - \frac{1}{T}\right)\right]}$$

41. Simplified Johnson-Lewin (4 parameters)[28,29]

$$B(T) = B_0 \cdot \frac{\exp\left(\frac{-E}{k_{\mathrm{B}}} \cdot \frac{1}{T}\right)}{1 + \frac{E}{E_{\mathrm{D}} - E} \cdot \exp\left[\frac{E_{\mathrm{D}}}{k_{\mathrm{B}}} \cdot \left(\frac{1}{T_{\mathrm{pk}}} - \frac{1}{T}\right)\right]}$$

42. Simplified extended Johnson-Lewin (5 parameters)[28–30]

$$B(T) = B_0 \cdot \frac{\exp\left(\frac{-E}{k_{\mathrm{B}}} \cdot \frac{1}{T}\right)}{a + \frac{E}{E_{\mathrm{D}} - E} \cdot \exp\left[\frac{E_{\mathrm{D}}}{k_{\mathrm{B}}} \cdot \left(\frac{1}{T_{\mathrm{pk}}} - \frac{1}{T}\right)\right]}$$

43. Kumaraswamy (5 parameters)[31]

$$B(T) = a \cdot b \cdot c \cdot \left(\frac{T - T_{\min}}{T_{\max} - T_{\min}}\right)^{b-1} \cdot \left[1 - \left(\frac{T - T_{\min}}{T_{\max} - T_{\min}}\right)^{b}\right]^{c-1}$$

44. Lactin I (3 parameters)[32]

$$B(T) = \exp(\rho \cdot T) - \exp\left(\rho \cdot T_{\max} - \frac{T_{\max} - T}{\Delta T}\right)$$

45. Lactin II (4 parameters)[32]

$$B(T) = \exp(\rho \cdot T) - \exp\left(\rho \cdot T_{\max} - \frac{T_{\max} - T}{\Delta T}\right) + \lambda$$

46. Linear-logistic (5 parameters)[33]

$$B(T) = a \cdot (T - T_{\min}) \cdot \frac{1 - \exp[-b \cdot (T_{\max} - T)]}{1 + \exp[-b \cdot (c - T)]}$$

47. Logan I (4 parameters)[34]

$$B(T) = \psi \cdot \left[\exp(\rho \cdot T) - \exp\left(\rho \cdot T_{\max} - \frac{T_{\max} - T}{\Delta T}\right)\right]$$

48. Logan II (5 parameters)[34]

$$B(T) = a \cdot \left[\frac{1}{1 + k \cdot \exp(-\rho \cdot T)} - \exp\left(-\frac{T_{\max} - T}{\Delta T}\right)\right]$$

49. Logan III (4 parameters)[35]

$$B(T) = \psi \cdot \left[\frac{T^2}{T^2 + D^2} - \exp\left(-\frac{T_{\max} - T}{\Delta T}\right)\right]$$

50. Mitchell-Angilletta (3 parameters)[36]

$$B(T) = \frac{a}{2 \cdot b} \cdot \left[1 + \cos\left(\frac{T - T_{\mathrm{pk}}}{b} \cdot \pi\right)\right]$$

51. Newbery (4 parameters)[37]

$$B(T) = a + b \cdot T + c \cdot [1 - \exp(d \cdot T^2)]$$

52. O'Neill (4 parameters)[38]

$$B(T) = B_{\mathrm{pk}} \cdot V^X \cdot \exp[X \cdot (1 - V)] \quad \textbf{where} \quad V = \frac{T_{\mathbf{max}} - T}{T_{\mathbf{max}} - T_{\mathbf{pk}}}, \quad W = (Q_{\mathbf{10}} - 1) \cdot (T_{\mathbf{max}} - T_{\mathbf{pk}}), \quad \textbf{and} \quad X = \frac{W^2 \cdot \left(1 + \sqrt{1 + \frac{40}{W}}\right)^2}{400}.$$

53. Second-order polynomial (3 parameters)

$$B(T) = a + b \cdot T + c \cdot T^2$$

54. Third-order polynomial (4 parameters)

$$B(T) = a + b \cdot T + c \cdot T^2 + d \cdot T^3$$

55. Fourth-order polynomial (5 parameters)

$$B(T) = a + b \cdot T + c \cdot T^2 + d \cdot T^3 + f \cdot T^4$$

56. Fifth-order polynomial (6 parameters)

$$B(T) = a + b \cdot T + c \cdot T^2 + d \cdot T^3 + f \cdot T^4 + g \cdot T^5$$

57. Ratkowsky (4 parameters)[39]

$$B(T) = [a \cdot (T - T_{\text{min}}) \cdot (1 - \exp(b \cdot (T - T_{\text{max}})))]^2$$

58. Régnière (6 parameters)[40]

$$B(T) = \psi \cdot \left[\exp(\rho \cdot (T - T_{\text{min}})) - \frac{T_{\text{max}} - T}{T_{\text{max}} - T_{\text{min}}} \cdot \exp\left(-\rho \cdot \frac{T - T_{\text{min}}}{\Delta T_{\text{low}}}\right) - \frac{T - T_{\text{min}}}{T_{\text{max}} - T_{\text{min}}} \cdot \exp\left(\rho \cdot (T_{\text{max}} - T_{\text{min}}) - \frac{T_{\text{max}} - T}{\Delta T_{\text{high}}}\right)\right]$$

59. Rezende-Bozinovic (4 parameters)[41]

$$B(T) = \begin{cases} B_0 \cdot \exp\left(\dfrac{T \cdot \ln Q_{10}}{10}\right) & \text{for } T \leq T_{\text{th}} \\ B_0 \cdot \exp\left(\dfrac{T \cdot \ln Q_{10}}{10}\right) \cdot [1 - d \cdot (T - T_{\text{th}})^2] & \text{for } T > T_{\text{th}} \end{cases}$$

60. Rice Clock (6 parameters)[42]

$$B(T) = \begin{cases} B_{\mathrm{pk}} \cdot \Phi & \text{for } \Phi \leq 1 \\ B_{\mathrm{pk}} & \text{for } \Phi > 1 \end{cases} \quad \textbf{where} \quad \Phi = \left(\frac{T - T_{\mathbf{min}}}{T_{\mathbf{pk}} - T_{\mathbf{min}}}\right)^{a} \cdot \left(\frac{T_{\mathbf{max}} - T}{T_{\mathbf{max}} - T_{\mathbf{pk}}}\right)^{b}$$

61. Ritchie (4 parameters)[43]

$$B(T) = R \cdot d_0 \cdot \exp\left(\frac{-E_{\mathrm{D}}}{R \cdot T}\right) \cdot \left(\frac{\Delta E}{R \cdot T} + a\right)$$

62. Ross-Ratkowsky (5 parameters)[44]

$$B(T) = \frac{a \cdot T \cdot \exp\left(\frac{-\Delta H_{\mathrm{A}}^{\ddagger}}{R \cdot T}\right)}{1 + \exp\left[-n \cdot \frac{\Delta H^{*} - 18.1 \cdot T + \Delta C_{\mathrm{p}} \cdot \left(T - 373.6 - T \cdot \ln \frac{T}{385.2}\right)}{R \cdot T}\right]}$$

63. Ruiz (4 parameters)[45]

$$B(T) = B_0 + \Delta B_{\mathrm{pk}} \cdot \exp[-a \cdot (T - T_{\mathrm{pk}})^2]$$

64. Sharpe-Schoolfield (6 parameters)[46]

$$B(T) = \frac{B_0 \cdot \frac{T}{T_{\mathrm{ref}}} \cdot \exp\left[\frac{\Delta H_{\mathrm{A}}^{\neq}}{R} \cdot \left(\frac{1}{T_{\mathrm{ref}}} - \frac{1}{T}\right)\right]}{1 + \exp\left[\frac{\Delta H_{\mathrm{L}}}{R} \cdot \left(\frac{1}{T_{\mathrm{L50}}} - \frac{1}{T}\right)\right] + \exp\left[\frac{\Delta H_{\mathrm{H}}}{R} \cdot \left(\frac{1}{T_{\mathrm{H50}}} - \frac{1}{T}\right)\right]}$$

65. Extended Sharpe-Schoolfield (7 parameters)[30,46]

$$B(T) = \frac{B_0 \cdot \frac{T}{T_{\text{ref}}} \cdot \exp\left[\frac{\Delta H_{\text{A}}^{\neq}}{R} \cdot \left(\frac{1}{T_{\text{ref}}} - \frac{1}{T}\right)\right]}{a + \exp\left[\frac{\Delta H_{\text{L}}}{R} \cdot \left(\frac{1}{T_{\text{L50}}} - \frac{1}{T}\right)\right] + \exp\left[\frac{\Delta H_{\text{H}}}{R} \cdot \left(\frac{1}{T_{\text{H50}}} - \frac{1}{T}\right)\right]}$$

66. Simplified Sharpe-Schoolfield (6 parameters)[46]

$$B(T) = \frac{B_0 \cdot \exp\left[\frac{\Delta H_{\text{A}}^{\neq}}{R} \cdot \left(\frac{1}{T_{\text{ref}}} - \frac{1}{T}\right)\right]}{1 + \exp\left[\frac{\Delta H_{\text{L}}}{R} \cdot \left(\frac{1}{T_{\text{L50}}} - \frac{1}{T}\right)\right] + \exp\left[\frac{\Delta H_{\text{H}}}{R} \cdot \left(\frac{1}{T_{\text{H50}}} - \frac{1}{T}\right)\right]}$$

67. Simplified extended Sharpe-Schoolfield (7 parameters)[30,46]

$$B(T) = \frac{B_0 \cdot \exp\left[\frac{\Delta H_{\text{A}}^{\neq}}{R} \cdot \left(\frac{1}{T_{\text{ref}}} - \frac{1}{T}\right)\right]}{a + \exp\left[\frac{\Delta H_{\text{L}}}{R} \cdot \left(\frac{1}{T_{\text{L50}}} - \frac{1}{T}\right)\right] + \exp\left[\frac{\Delta H_{\text{H}}}{R} \cdot \left(\frac{1}{T_{\text{H50}}} - \frac{1}{T}\right)\right]}$$

68. Simplified β type (3 parameters)[47]

$$B(T) = \rho \cdot \left(a - \frac{T}{10}\right) \cdot \left(\frac{T}{10}\right)^{b}$$

69. Skew-normal (4 parameters)[48]

$$B(T) = a \cdot \exp\left[\frac{-(T - z)^2}{\sigma^2}\right] \cdot \left[1 + \text{erf}\left(\frac{\lambda \cdot (T - z)}{\sigma}\right)\right]$$

70. Stevenson (6 parameters)[49]

$$B(T) = a \cdot \frac{1 - \exp[b \cdot (T - T_{\text{max}})]}{1 + c \cdot \exp[-d \cdot (T - T_{\text{min}})]}$$

71. Stinner (4 parameters)[50]

$$B(T) = \begin{cases} B_{\text{pk}} \cdot \dfrac{1 + \exp(a + b \cdot T_{\text{pk}})}{1 + \exp(a + b \cdot T)} & \text{for } T \leq T_{\text{pk}} \\ B_{\text{pk}} \cdot \dfrac{1 + \exp(a + b \cdot T_{\text{pk}})}{1 + \exp[a + b \cdot (2 \cdot T_{\text{pk}} - T)]} & \text{for } T > T_{\text{pk}} \end{cases}$$

72. Taylor-Sexton (3 parameters)[51]

$$B(T) = B_{\text{pk}} \cdot \frac{-(T - T_{\text{min}})^4 + 2 \cdot (T - T_{\text{min}})^2 \cdot (T_{\text{pk}} - T_{\text{min}})^2}{(T_{\text{pk}} - T_{\text{min}})^4}$$

73. Thomas I (4 parameters)[52]

$$B(T) = a \cdot \exp(b \cdot T) \cdot \left[1 - \left(\frac{T - z}{\frac{w}{2}}\right)^2\right]$$

This model is mathematically equivalent to that fitted by Norberg, 2004[53].

74. Thomas II (5 parameters)[54]

$$B(T) = a \cdot \exp(b \cdot T) - [c + d \cdot \exp(f \cdot T)]$$

75. Thornton-Lessem (6 parameters)[55]

$$B(T) = a \cdot \frac{K_1 \cdot \exp\left[\frac{T - T_1}{T_2 - T_1} \cdot \ln\frac{0.98 \cdot (1 - K_1)}{K_1 \cdot (1 - 0.98)}\right]}{1 + K_1 \cdot \left[\exp\left(\frac{T - T_1}{T_2 - T_1} \cdot \ln\frac{0.98 \cdot (1 - K_1)}{K_1 \cdot (1 - 0.98)}\right) - 1\right]} \cdot \frac{K_4 \cdot \exp\left[\frac{T_3 - T}{T_3 - T_2} \cdot \ln\frac{0.98 \cdot (1 - K_4)}{K_4 \cdot (1 - 0.98)}\right]}{1 + K_4 \cdot \left[\exp\left(\frac{T_3 - T}{T_3 - T_2} \cdot \ln\frac{0.98 \cdot (1 - K_4)}{K_4 \cdot (1 - 0.98)}\right) - 1\right]}$$

76. Tomlinson-Menz (4 parameters)[56]

$$B(T) = a \cdot [\exp(b \cdot T) - \exp(c - T) - \exp(T - d)]$$

77. Tomlinson-Phillips (3 parameters)[57]

$$B(T) = a \cdot [\exp(b \cdot T) - \exp(T - c)]$$

78. Van't Hoff (4 parameters)[58]

$$B(T) = a \cdot \exp\left(\frac{-b}{T}\right) \cdot T^{c} \cdot \exp(d \cdot T)$$

79. Wang-Engel (4 parameters)[59]

$$B(T) = B_{\mathrm{pk}} \cdot \frac{2 \cdot (T - T_{\min})^{a} \cdot (T_{\mathrm{pk}} - T_{\min})^{a} - (T - T_{\min})^{2 \cdot a}}{(T_{\mathrm{pk}} - T_{\min})^{2 \cdot a}}$$

80. Wang-Lan-Ding (7 parameters)[60]

$$B(T) = k \cdot \frac{[1 - \exp(-a \cdot (T - T_{\min}))] \cdot [1 - \exp(b \cdot (T - T_{\max}))]}{1 + \exp[-d \cdot (T - T_0)]}$$

81. Warren-Dreyer (3 parameters)[61]

$$B(T) = B_{\mathrm{pk}} \cdot \exp\left[-0.5 \cdot \left(\frac{\ln\frac{T}{T_{\mathrm{pk}}}}{a}\right)^2\right]$$

82. Weibull (4 parameters)[62]

$$B(T) = B_{\mathrm{pk}} \cdot \left(\frac{c-1}{c}\right)^{(1-c)/c} \cdot \left[\frac{T - T_{\mathrm{pk}}}{b} + \left(\frac{c-1}{c}\right)^{1/c}\right]^{c-1} \cdot \exp\left[-\left(\frac{T - T_{\mathrm{pk}}}{b} + \left(\frac{c-1}{c}\right)^{1/c}\right)^{c} + \frac{c-1}{c}\right]$$

83. Yan-Hunt (4 parameters)[63]

$$B(T) = B_{\mathrm{pk}} \cdot \frac{T_{\max} - T}{T_{\max} - T_{\mathrm{pk}}} \cdot \left(\frac{T - T_{\min}}{T_{\mathrm{pk}} - T_{\min}}\right)^{\frac{T_{\mathrm{pk}} - T_{\min}}{T_{\max} - T_{\mathrm{pk}}}}$$

Supplementary References

1. Allahyari, H. *Decision making with degree-day in control program of Colorado potato beetle.* Ph.D. thesis, University of Tehran, Tehran, Iran (2005).

2. Kontodimas, D. C., Eliopoulos, P. A., Stathas, G. J. & Economou, L. P. Comparative temperature-dependent development of *Nephus includens* (Kirsch) and *Nephus bisignatus* (Boheman) (Coleoptera: Coccinellidae) preying on *Planococcus citri* (Risso) (Homoptera: Pseudococcidae): evaluation of a linear and various nonlinear models using specific criteria. *Environ. Entomol.* **33**, 1–11 (2004).

3. Asbury, D. A. & Angilletta Jr, M. J. Thermodynamic effects on the evolution of performance curves. *Am. Nat.* **176**, E40–E49 (2010).

4. Ashrafi, R. *et al.* Broad thermal tolerance is negatively correlated with virulence in an opportunistic bacterial pathogen. *Evol. Appl.* **11**, 1700–1714 (2018).

5. Atkin, O. K., Bruhn, D. & Tjoelker, M. G. Response of plant respiration to changes in temperature: mechanisms and consequences of variations in Q_{10} values and acclimation. In *Plant Respiration*, 95–135 (Springer, 2005).

6. Olsen, J. K., McMahon, C. R. & Hammer, G. L. Prediction of sweet corn phenology in subtropical environments. *Agron. J.* **85**, 410–415 (1993).

7. Torabi, B., Archontoulis, S. V. & Hoogenboom, G. A new function for prediction of biological processes response to temperature. *Int. J. Plant Prod.* **14**, 9–22 (2020).

8. Boatman, T. G., Lawson, T. & Geider, R. J. A key marine diazotroph in a changing ocean: the interacting effects of temperature, CO_2 and light on the growth of *Trichodesmium erythraeum* IMS101. *PLoS ONE* **12**, e0168796 (2017).

9. Briere, J.-F., Pracros, P., Le Roux, A.-Y. & Pierre, J.-S. A novel rate model of temperature-dependent development for arthropods. *Environ. Entomol.* **28**, 22–29 (1999).

10. Cruz-Loya, M. *et al.* Antibiotics shift the temperature response curve of *Escherichia coli* growth. *mSystems* **6**, e00228–21 (2021).

11. Lobry, J. R., Rosso, L. & Flandrois, J.-P. A FORTRAN subroutine for the determination of parameter confidence limits in non-linear models. *Binary* **3**, 86–93 (1991).

12. Rosso, L., Lobry, J. R. & Flandrois, J. P. An unexpected correlation between cardinal temperatures of microbial growth highlighted by a new model. *J. Theor. Biol.* **162**, 447–463 (1993).

13. Soltani, A., Hammer, G. L., Torabi, B., Robertson, M. J. & Zeinali, E. Modeling chickpea growth and development: phenological development. *Field Crops Res.* **99**, 1–13 (2006).

14. Deutsch, C. A. *et al.* Impacts of climate warming on terrestrial ectotherms across latitude. *Proc. Natl. Acad. Sci. U.S.A.* **105**, 6668–6672 (2008).

15. Krenek, S., Berendonk, T. U. & Petzoldt, T. Thermal performance curves of *Paramecium caudatum*: a model selection approach. *Eur. J. Protistol.* **47**, 124–137 (2011).

16. DeLong, J. P. *et al.* The combined effects of reactant kinetics and enzyme stability explain the temperature dependence of metabolic rates. *Ecol. Evol.* **7**, 3940–3950 (2017).

17. Eubank, W. P., Atmar, J. W. & Ellington, J. J. The significance and thermodynamics of fluctuating versus static thermal environments on *Heliothis zea* egg development rates. *Environ. Entomol.* **2**, 491–496 (1973).

18. Finstad, A. G. & Jonsson, B. Effect of incubation temperature on growth performance in Atlantic salmon. *Mar. Ecol. Prog. Ser.* **454**, 75–82 (2012).

19. Angilletta Jr, M. J. Estimating and comparing thermal performance curves. *J. Therm. Biol.* **31**, 541–545 (2006).

20. Phillips, B. L., Llewelyn, J., Hatcher, A., Macdonald, S. & Moritz, C. Do evolutionary constraints on thermal performance manifest at different organizational scales? *J. Evol. Biol.* **27**, 2687–2694 (2014).

21. Woods, H. A., Kingsolver, J. G., Fey, S. B. & Vasseur, D. A. Uncertainty in geographical estimates of performance and fitness. *Methods Ecol. Evol.* **9**, 1996–2008 (2018).

22. Frazier, M. R., Huey, R. B. & Berrigan, D. Thermodynamics constrains the evolution of insect population growth rates: "warmer is better". *Am. Nat.* **168**, 512–520 (2006).

23. Hinshelwood, C. N. *The chemical kinetics of the bacterial cell* (Clarendon Press, Oxford, 1946).

24. Hobbs, J. K. *et al.* Change in heat capacity for enzyme catalysis determines temperature dependence of enzyme catalyzed rates. *ACS Chem. Biol.* **8**, 2388–2393 (2013).

25. Huey, R. B. & Stevenson, R. D. Integrating thermal physiology and ecology of ectotherms: a discussion of approaches. *Amer. Zool.* **19**, 357–366 (1979).

26. Janisch, E. Über die Temperaturabhängigkeit biologischer Vorgänge und ihre kurvenmäßige Analyse. *Pflüger's Arch. Physiol.* **209**, 414–436 (1925).

27. Jöhnk, K. D. *et al.* Summer heatwaves promote blooms of harmful cyanobacteria. *Glob. Chang. Biol.* **14**, 495–512 (2008).

28. Johnson, F. H. & Lewin, I. The growth rate of *E. coli* in relation to temperature, quinine and coenzyme. *J. Cell. Comp. Physiol.* **28**, 47–75 (1946).

29. Dell, A. I., Pawar, S. & Savage, V. M. Systematic variation in the temperature dependence of physiological and ecological traits. *Proc. Natl. Acad. Sci. U.S.A.* **108**, 10591–10596 (2011).

30. Guenther, A. Seasonal and spatial variations in natural volatile organic compound emissions. *Ecol. Appl.* **7**, 34–45 (1997).

31. Tittes, S. B., Walker, J. F., Torres-Martínez, L. & Emery, N. C. Grow where you thrive, or where only you can survive? An analysis of performance curve evolution in a clade with diverse habitat affinities. *Am. Nat.* **193**, 530–544 (2019).

32. Lactin, D. J., Holliday, N. J., Johnson, D. L. & Craigen, R. Improved rate model of temperature-dependent development by arthropods. *Environ. Entomol.* **24**, 68–75 (1995).

33. Hui, T. Y., Yuan, F. L., Bonebrake, T. C. & Williams, G. A. Multifunctional behaviour in a sandy shore crab enhances performance in extreme intertidal environments. *Oecologia* **189**, 79–89 (2019).

34. Logan, J. A., Wollkind, D. J., Hoyt, S. C. & Tanigoshi, L. K. An analytic model for description of temperature dependent rate phenomena in arthropods. *Environ. Entomol.* **5**, 1133–1140 (1976).

35. Hilbert, D. & Logan, J. Empirical model of nymphal development for the migratory grasshopper, *Melanoplus sanguinipes* (Orthoptera: Acrididae). *Environ. Entomol.* **12**, 1–5 (1983).

36. Mitchell, W. A. & Angilletta Jr, M. J. Thermal games: frequency-dependent models of thermal adaptation. *Funct. Ecol.* **23**, 510–520 (2009).

37. Newbery, F., Ritchie, F., Gladders, P., Fitt, B. D. L. & Shaw, M. W. Inter-individual genetic variation in the temperature response of *Leptosphaeria* species pathogenic on oilseed rape. *Plant Pathol.* **69**, 1469–1481 (2020).

38. O'Neill, R. V., Goldstein, R. A., Shugart, H. H. & Mankin, J. B. Terrestrial ecosystem energy model. In *Eastern Deciduous Forest Biome memo report 72-19* (Environmental Sciences Division, Oak Ridge National Laboratory, Oak Ridge, TN, 1972).

39. Ratkowsky, D. A., Lowry, R. K., McMeekin, T. A., Stokes, A. N. & Chandler, R. E. Model for bacterial culture growth rate throughout the entire biokinetic temperature range. *J. Bacteriol.* **154**, 1222–1226 (1983).

40. Régnière, J., Powell, J., Bentz, B. & Nealis, V. Effects of temperature on development, survival and reproduction of insects: experimental design, data analysis and modeling. *J. Insect Physiol.* **58**, 634–647 (2012).

41. Rezende, E. L. & Bozinovic, F. Thermal performance across levels of biological organization. *Philos. Trans. R. Soc. Lond., B, Biol. Sci.* **374**, 20180549 (2019).

42. Gao, L., Jin, Z., Huang, Y. & Zhang, L. Rice clock model—a computer model to simulate rice development. *Agric. For. Meteorol.* **60**, 1–16 (1992).

43. Ritchie, M. E. Reaction and diffusion thermodynamics explain optimal temperatures of biochemical reactions. *Sci. Rep.* **8**, 1–10 (2018).

44. Ratkowsky, D. A., Olley, J. & Ross, T. Unifying temperature effects on the growth rate of bacteria and the stability of globular proteins. *J. Theor. Biol.* **233**, 351–362 (2005).

45. Ruiz, T. *et al.* The threshold elemental ratio of an ectotherm decreases then increases with rising temperature. *bioRxiv* (2019).

46. Schoolfield, R. M., Sharpe, P. J. H. & Magnuson, C. E. Non-linear regression of biological temperature-dependent rate models based on absolute reaction-rate theory. *J. Theor. Biol.* **88**, 719–731 (1981).

47. Damos, P. & Savopoulou-Soultani, M. Temperature-dependent bionomics and modeling of *Anarsia lineatella* (Lepidoptera: Gelechiidae) in the laboratory. *J. Econ. Entomol.* **101**, 1557–1567 (2008).

48. Urban, M. C., Tewksbury, J. J. & Sheldon, K. S. On a collision course: competition and dispersal differences create no-analogue communities and cause extinctions during climate change. *Proc. R. Soc. B* **279**, 2072–2080 (2012).

49. Stevenson, R. D., Peterson, C. R. & Tsuji, J. S. The thermal dependence of locomotion, tongue flicking, digestion, and oxygen consumption in the wandering garter snake. *Physiol. Zool.* **58**, 46–57 (1985).

50. Stinner, R. E., Gutierrez, A. P. & Butler, G. D. An algorithm for temperature-dependent growth rate simulation. *Can. Ent.* **106**, 519–524 (1974).

51. Taylor, S. E. & Sexton, O. J. Some implications of leaf tearing in Musaceae. *Ecology* **53**, 143–149 (1972).

52. Thomas, M. K., Kremer, C. T., Klausmeier, C. A. & Litchman, E. A global pattern of thermal adaptation in marine phytoplankton. *Science* **338**, 1085–1088 (2012).

53. Norberg, J. Biodiversity and ecosystem functioning: a complex adaptive systems approach. *Limnol. Oceanogr.* **49**, 1269–1277 (2004).

54. Thomas, M. K. *et al.* Temperature–nutrient interactions exacerbate sensitivity to warming in phytoplankton. *Glob. Chang. Biol.* **23**, 3269–3280 (2017).

55. Thornton, K. W. & Lessem, A. S. A temperature algorithm for modifying biological rates. *Trans. Am. Fish. Soc.* **107**, 284–287 (1978).

56. Tomlinson, S. & Menz, M. H. M. Does metabolic rate and evaporative water loss reflect differences in migratory strategy in sexually dimorphic hoverflies? *Comp. Biochem. Physiol. A Mol. Integr. Physiol.* **190**, 61–67 (2015).

57. Tomlinson, S. & Phillips, R. D. Differences in metabolic rate and evaporative water loss associated with sexual dimorphism in thynnine wasps. *J. Insect Physiol.* **78**, 62–68 (2015).

58. Portner, H., Bugmann, H. & Wolf, A. Temperature response functions introduce high uncertainty in modelled carbon stocks in cold temperature regimes. *Biogeosciences* **7**, 3669–3684 (2010).

59. Wang, E. & Engel, T. Simulation of phenological development of wheat crops. *Agric. Syst.* **58**, 1–24 (1998).

60. Wang, R. S., Lan, Z. X. & Ding, Y. Q. Studies on mathematical models of the relationship between insect development and temperature. *Acta Ecol. Sin.* **2**, 47–57 (1982).

61. Warren, C. R. & Dreyer, E. Temperature response of photosynthesis and internal conductance to CO_2: results from two independent approaches. *J. Exp. Bot.* **57**, 3057–3067 (2006).

62. Shi, P. & Ge, F. A comparison of different thermal performance functions describing temperature-dependent development rates. *J. Therm. Biol.* **35**, 225–231 (2010).

63. Yan, W. & Hunt, L. A. An equation for modelling the temperature response of plants using only the cardinal temperatures. *Ann. Bot.* **84**, 607–614 (1999).
