# Supplementary material for: No universal mathematical model for thermal performance curves across traits and taxonomic groups
Source: Nat Commun. 2024 Oct 14;15:8855. doi: 10.1038/s41467-024-53046-2 (PMC11473535; doi:10.1038/s41467-024-53046-2)
Supplement: Supplementary file 3 — Reporting Summary [file 41467_2024_53046_MOESM3_ESM.pdf]

Corresponding author(s): Dimitrios - Georgios Kontopoulos

Last updated by author(s): Sep 16, 2024

Reporting Summary

Nature Portfolio wishes to improve the reproducibility of the work that we publish. This form provides structure for consistency and transparency in reporting. For further information on Nature Portfolio policies, see our Editorial Policies and the Editorial Policy Checklist.

Statistics

For all statistical analyses, confirm that the following items are present in the figure legend, table legend, main text, or Methods section.

n/a	Confirmed	
☐	☒	The exact sample size (*n*) for each experimental group/condition, given as a discrete number and unit of measurement
☐	☒	A statement on whether measurements were taken from distinct samples or whether the same sample was measured repeatedly
☒	☐	The statistical test(s) used AND whether they are one- or two-sided<br>*Only common tests should be described solely by name; describe more complex techniques in the Methods section.*
☐	☒	A description of all covariates tested
☒	☐	A description of any assumptions or corrections, such as tests of normality and adjustment for multiple comparisons
☐	☒	A full description of the statistical parameters including central tendency (e.g. means) or other basic estimates (e.g. regression coefficient) AND variation (e.g. standard deviation) or associated estimates of uncertainty (e.g. confidence intervals)
☒	☐	For null hypothesis testing, the test statistic (e.g. *F*, *t*, *r*) with confidence intervals, effect sizes, degrees of freedom and *P* value noted<br>*Give P values as exact values whenever suitable.*
☒	☐	For Bayesian analysis, information on the choice of priors and Markov chain Monte Carlo settings
☒	☐	For hierarchical and complex designs, identification of the appropriate level for tests and full reporting of outcomes
☒	☐	Estimates of effect sizes (e.g. Cohen's *d*, Pearson's *r*), indicating how they were calculated

Our web collection on statistics for biologists contains articles on many of the points above.

Software and code

Policy information about availability of computer code

Data collection	No software was used for data collection.
Data analysis	We used the following R packages: ComplexHeatmap (v.2.12.1), ggtree (v. 2.2.4), nls.multstart (v. 1.2.0), and partykit (v. 1.2-17). R scripts for the analyses of this study have been deposited in a GitHub repository (https://github.com/dgkontopoulos/Kontopoulos_et_al_83_TPC_models_2024) and archived on Zenodo (https://doi.org/10.5281/zenodo.12608191).

For manuscripts utilizing custom algorithms or software that are central to the research but not yet described in published literature, software must be made available to editors and reviewers. We strongly encourage code deposition in a community repository (e.g. GitHub). See the Nature Portfolio guidelines for submitting code & software for further information.

Data

Policy information about availability of data

All manuscripts must include a data availability statement. This statement should provide the following information, where applicable:

- Accession codes, unique identifiers, or web links for publicly available datasets
- A description of any restrictions on data availability
- For clinical datasets or third party data, please ensure that the statement adheres to our policy

The thermal performance datasets included in this study are available from Figshare: https://doi.org/10.6084/m9.figshare.24106161.v3. These datasets were collected from numerous previous studies or from the Biotraits database. Source data for all figures are also provided as a Source Data File.

Research involving human participants, their data, or biological material

Policy information about studies with human participants or human data. See also policy information about sex, gender (identity/presentation), and sexual orientation and race, ethnicity and racism.

Reporting on sex and gender	N/A
Reporting on race, ethnicity, or other socially relevant groupings	N/A
Population characteristics	N/A
Recruitment	N/A
Ethics oversight	N/A

Note that full information on the approval of the study protocol must also be provided in the manuscript.

Field-specific reporting

Please select the one below that is the best fit for your research. If you are not sure, read the appropriate sections before making your selection.

☐ Life sciences ☐ Behavioural & social sciences ☒ Ecological, evolutionary & environmental sciences

For a reference copy of the document with all sections, see nature.com/documents/nr-reporting-summary-flat.pdf

Ecological, evolutionary & environmental sciences study design

All studies must disclose on these points even when the disclosure is negative.

Study description	In ectotherms, the performance of physiological, ecological, and life-history traits is a unimodal function of temperature, called the "thermal performance curve". The shape of this curve can vary in many ways across traits, taxa, and environments. To quantify different aspects of this curve, numerous mathematical models have been used in the literature. Nevertheless, it remains unclear if certain models are more appropriate than others for particular traits, taxa, or sample sizes or if specific models generally outperform others, regardless of dataset. To fill this gap, we collect 83 mathematical models used in previous studies to estimate thermal performance curves. We then evaluate and compare their performance across 2,739 thermal performance datasets from taxa from all seven kingdoms and from more than 100 traits. Last, we employ multi-output conditional inference trees (an interpretable machine learning algorithm) to understand if there are well-defined scenarios in which certain models consistently outperform others.
Research sample	We collected thermal performance datasets for biological traits or individual enzymes from the literature (see the "Sources of thermal performance datasets" section). Additionally, in the data files uploaded to Figshare, we include a "citation" column for each dataset.
Sampling strategy	No new samples were obtained for this study. Given that the simplest models in our catalogue had three parameters, we only included thermal performance datasets with at least four experimental temperatures.
Data collection	We collected the thermal performance datasets from previous studies or from the Biotraits database.
Timing and spatial scale	N/A
Data exclusions	We only included thermal performance datasets with at least four experimental temperatures. We additionally removed datasets that were poorly fit by all of our 83 models (see Figure 2d and the Methods section).
Reproducibility	N/A
Randomization	We trained a series of multi-output conditional inference trees to predict the AICc weights of our 83 models based on a) trait or taxonomic information, b) the shape of the thermal performance curve, or c) the sampling resolution. To this end, we randomly split our data into a training subset (80%) and a testing subset (20%).
Blinding	N/A

Did the study involve field work? ☐ Yes ☒ No

Reporting for specific materials, systems and methods

We require information from authors about some types of materials, experimental systems and methods used in many studies. Here, indicate whether each material, system or method listed is relevant to your study. If you are not sure if a list item applies to your research, read the appropriate section before selecting a response.

Materials & experimental systems

n/a	Involved in the study
☒	☐ Antibodies
☒	☐ Eukaryotic cell lines
☒	☐ Palaeontology and archaeology
☒	☐ Animals and other organisms
☒	☐ Clinical data
☒	☐ Dual use research of concern
☒	☐ Plants

Methods

n/a	Involved in the study
☒	☐ ChIP-seq
☒	☐ Flow cytometry
☒	☐ MRI-based neuroimaging

Plants

Seed stocks	N/A
Novel plant genotypes	N/A
Authentication	N/A
